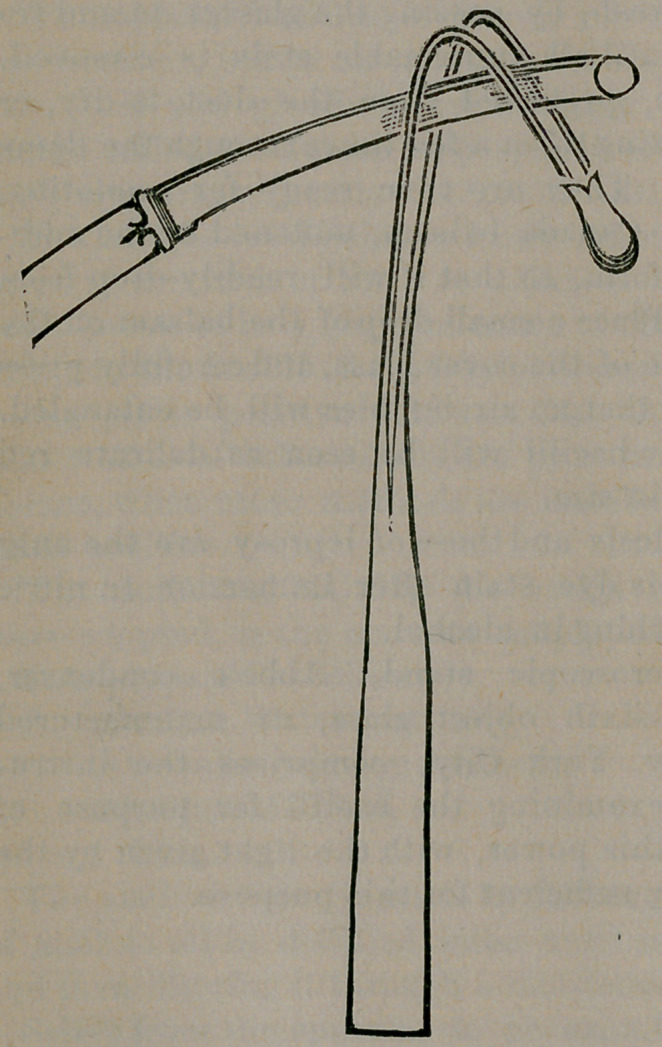# New Operation and Instrument for Fistula in Ano

**Published:** 1885-09

**Authors:** J. A. Gibson

**Affiliations:** Foster, Texas


### NEW OPERATION AND INSTRUMENT FOR FISTULA IN ANO.

*By J. A. Gibson, M. D., Foster, Texas.*

For Daniel's Texas Medical Journal.

ON the 30th of March last, assisted by Dr. K. L. Harris, I operated in the following manner, for complete fistula in ano: I took a small silver wire, and doubling it about the middle, introduced it into the bowel through the fistula; brought out the end, and putting both ends together, and making traction, I cut down by the side of the wire. This was accomplished with less pain, I think, than would have been inflicted by operating in the usual way with a grooved director—straight,–brought forcibly out the rectum by the point of the finger.

A better plan still, in my judgment, would be to dilate the sphincter well, first; then with the wire in situ, cut from the inner opening, outward, putting a probe pointed bistoury between the wires as suggested by Dr. Q.C.Smith,of Austin, i.e., the wire should be doubled twice.

Probably a still better plan would be to use an instrument similar to the one shown in the accompanying cut. Dilate the sphincter well,—introduce the probe point of the instrument into the inner opening, bringing it out at the outer opening, if the fistula be complete ; then pass the blade of a probe pointed knife between the wires, cutting edge downward, as shown in the cut, and cut downward and outward, dividing the whole intervening tissues at one sweep. The advantages of such an instrument over a straight, grooved director which is introduced from without, into the gut, through perhaps a tortuous opening, and then forcibly pulled out at the anus, must be obvious. In case of incomplete fistula, where it opens only inwardly, such an instrument would be very serviceable.

## Figures and Tables

**Figure f1:**